# Application of RAD Sequencing for Evaluating the Genetic Diversity of Domesticated *Panax notoginseng* (Araliaceae)

**DOI:** 10.1371/journal.pone.0166419

**Published:** 2016-11-15

**Authors:** Yuezhi Pan, Xueqin Wang, Guiling Sun, Fusheng Li, Xun Gong

**Affiliations:** 1 Key Laboratory for Plant Diversity and Biogeography of East Asia, Kunming Institute of Botany, Chinese Academy of Sciences, Kunming, China; 2 College of Agronomy and Biotechnology, Yunnan Agricultural University, Kunming, China; 3 College of Life Science and Agronomy, Zhoukou Normal University, Zhoukou, China; 4 Yunnan Key Laboratory for Wild Plant Resources, Kunming, China; National Cheng Kung University, TAIWAN

## Abstract

*Panax notoginseng*, a traditional Chinese medicinal plant, has been cultivated and domesticated for approximately 400 years, mainly in Yunnan and Guangxi, two provinces in southwest China. This species was named according to cultivated rather than wild individuals, and no wild populations had been found until now. The genetic resources available on farms are important for both breeding practices and resource conservation. In the present study, the recently developed technology RADseq, which is based on next-generation sequencing, was used to analyze the genetic variation and differentiation of *P*. *notoginseng*. The nucleotide diversity and heterozygosity results indicated that *P*. *notoginseng* had low genetic diversity at both the species and population levels. Almost no genetic differentiation has been detected, and all populations were genetically similar due to strong gene flow and insufficient splitting time. Although the genetic diversity of *P*. *notoginseng* was low at both species and population levels, several traditional plantations had relatively high genetic diversity, as revealed by the *H*_e_ and *π* values and by the private allele numbers. These valuable genetic resources should be protected as soon as possible to facilitate future breeding projects. The possible geographical origin of Sanqi domestication was discussed based on the results of the genetic diversity analysis.

## Introduction

Crop species were first domesticated from their wild relatives approximately 10000 years ago [1]. Cornille et al[2] defined domesticated species as those segments of evolutionary lineages that diverge from their wild progenitors in response to artificial selection pressure and human control over reproduction. Crop domestication can lead to dramatic changes in agronomic traits. At the same time, the genetic bottleneck that occurs during this process can reduce the genetic diversity in cultivated plants and lead to a loss of genetic variation relative to the species’ wild ancestors [1,3]. Understanding the makeup and distribution of this genetic diversity has been our priority as we consider the process of crop genetic resources conservation and improvement. The assessment of the level and patterns of crop genetic diversity will also be helpful for estimating any possible loss of genetic diversity during conservation programs. Moreover, this assessment will be helpful for evaluating the effects of evolutionary forces (mutation, natural selection, gene flow and genetic drift) on population properties, such as effective population size, breeding systems, population structure, and dispersal mechanisms[4–6].

*Panax notoginseng*(Burkill) F. H. Chen ex C. Y. Wu et K M. Feng, commonly known as “Sanqi” in China, is a diploid (2n = 2x = 24) species[7]that belongs to the family Araliaceae and has a genome of approximately 2400 Mb in size [8]. It is a traditional Chinese medicinal plant that is widely used for cardiovascular diseases and has been domesticated and cultivated for approximately 400 years [9]. However, the serious root rot disease caused by pathogens limits the production of this herb [10]. Phylogenetic analysis confirmed its taxonomic position in the genus *Panax* [11,12]; however, this taxon was initially named according to cultivated rather than wild individuals, and no wild populations had ever been found until now [13].The genetic resources available in the farms are of the utmost importance for both breeding practices and resource conservation. A sustainable Sanqi-growing industry will rely on the access to and use of Sanqi’s genetic diversity to develop improved disease-resistant cultivars through marker-assisted breeding, genome-wide association studies (GWAS) and genomic selection (GS) [14,15]. Well-powered GWAS and GS require a genome-wide assessment of genetic diversity and population structure [14]. In addition, genetic management for the remnant Sanqi resources requires an assessment of the genetic diversity pattern.

*Panax notoginseng*is cultivated in some plantations of the Wenshan Autonomous Prefecture of Yunnan province and the Jingxi County of the Guangxi Zhuang Autonomous Region of China. Cultivated *P*. *notoginseng* displays a wide range of morphological diversity, such as white-yellow or dark red tuberous roots, green, dark red or mixed color stems, red or yellow fruits [16]. However, it exhibits low genetic diversity at the species level compared to a wild relative, *P*. *stipuleanatus*, as evidenced by ITS sequencing and AFLP polymorphism analysis [17]. No sequence variation in the ITS segment was detected among 24 individuals of *P*. *notoginseng* from three populations, and nine sites (1.30%) were variable in 51 accessions sampled from eight populations of *P*. *stipuleanatus*. The percentage of AFLP polymorphic sites was 76.9% in *P*. *notoginseng* and 96.5% in *P*. *stipuleanatus* [17]. *P*. *notoginseng* also harbored less DNA variation than did its two cultivated tetraploid relatives, *P*. *ginseng* and *P*. *quinquefolius*, as revealed by the screening of 36 single copy nuclear loci [18].This type of comparison of closely related species can potentially reveal the processes by which genetic diversity has recently or historically been altered. However, there is no guarantee that the mutation rate of a locus in one species will match that of another, which makes interspecific comparisons very challenging [19]. In addition, the data used in the *P*. *notoginseng* studies mentioned above were not sufficient for fully assessing the genetic structure and diversity, especially at the population level.

Next-generation sequencing (NGS) technology provides the opportunity to generate large-scale molecular marker data to study genetic diversity at a much higher resolution. Restriction-site associated DNA (RAD) sequencing is a method based on NGS technology that can create a reduced representation of the genome and identify thousands of genetic markers that are randomly distributed across the target genome. It promises to generate high-resolution population genomic data for model and non-model organisms [20]. For RAD sequencing, genomic DNA is digested by using a restriction enzyme such as *EcoR*I or *Sbf*I, or a combination of two enzymes, and is then sequenced from the restriction sites to yield a vast number of short reads [20–23]. RAD sequencing has been successfully applied to generate genome-wide SNP data to address questions in population genomics, phylogenetics and speciation studies [21,24–29]. Bioinformatic tools such as ***Stacks*** [30,31] and ***pyRAD*** can greatly [32] facilitate the analysis of the RAD short reads.

Sanqi currently faces severe pathogen pressures, and long-term sustainability projects and associated medical industries will rely on the exploitation of the existing natural genetic diversity. The specific objectives of the present study were to determine the genetic diversity, population divergence and structure at both the species and population levels by using RAD sequencing technology. The generated knowledge would be beneficial to breeding and germplasm conservation efforts of this medicinal crop.

## Materials and Methods

### Plant materials

**Fig 1:**
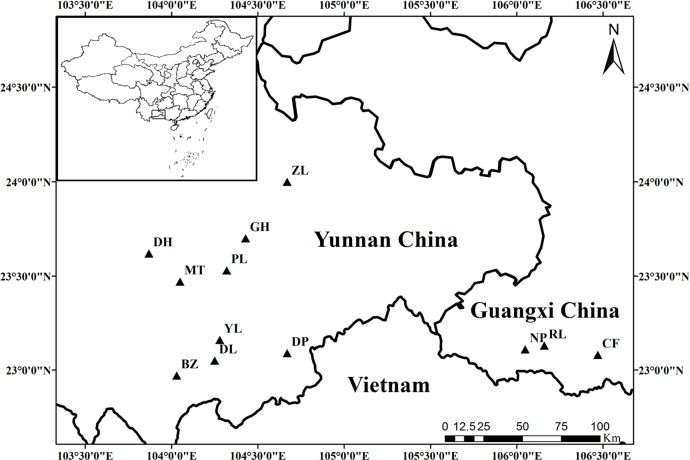
Sample locations of *Panax notoginseng*.

The materials included 36 samples from 12 plantations. Twenty-seven accessions were obtained from nine populations that were distributed in five Yunnan counties, and nine accessions were obtained from three populations distributed in the Jingxi county of Guangxi (Fig 1 and S1 Table). Three samples were randomly collected from each plantation. No specific permissions were required during the sample collection.

### Creation and sequencing of the RAD libraries and sequence analysis

Total genomic DNA was extracted from silica gel-dried leaf material using a modified CTAB procedure [33]. The genomic DNA samples were digested with *EcoR*I, and 36 RAD sequencing libraries were prepared according to the methods described previously [21,34]. In brief, the libraries were prepared following DNA digestion using the *EcoR*I enzyme, P1 adapter/barcode ligation and DNA purification, size selection, P2 adapter ligation and RAD tag amplification. Single-end sequencing was aimed to produce approximately 1,000 Mb raw data for each library using Illumina HiSeq2000. The steps mentioned above were carried out by Majorbio Pharm Technology Co., Ltd., Shanghai. The raw sequence data have been deposited in the National Center for Biotechnology Information (NCBI) Sequence-Read Archive (SRA) database with the accession numbers SRR3123274, SRR3123435—SRR3123442, SRR3123444, SRR3123447 and SRR3123450.

The raw data were analyzed *de novo* using the ***Stacks1*.*0*** pipeline [31,35]to reconstruct loci. The ***process_radtags*** program was used to demultiplex and sort the raw data according to the barcodes used in each sample. During this process, the adapter contamination was filtered out, and the raw reads with low average quality score bases (phred score ≤ 10) were discarded. The clean data for each sample were grouped into loci using ***ustacks***with a stack depth parameter (-m) of 5, a mismatch parameter (-M) of 2, and maximum stacks allowed per locus (—max_locus_stacks) of 3. The loci data of all of the samples were merged into a catalog using ***cstacks***, and then the loci of each sample were matched against the catalog so as to determine the allele status in each sample using ***sstacks***. To evaluate the genetic diversity of *P*. *notoginseng* at the species level using ***populations***, we treated all 36 samples as a whole population. To include a locus in this analysis, we required it to be present in at least 67% of the samples. When we evaluated the genetic diversity at the population level, we treated each plantation as a population and required a locus to be present in all individuals (r = 1) in at least six populations (p = 6).

Population genetic statistics, including the private allele number, heterozygosity (*H*), nucleotide diversity (*π*) and Wright’s F statistics *F*_IS_ and *F*_ST_, were calculated for every SNP using the ***populations*** program in ***Stacks***. For bi-allelic SNP markers, *π* is a useful overall measure of genetic diversity in a population, and the F statistic measures the distribution of genetic variation within and among populations [30,36]. To test whether there is a hidden population structure within each population, we examined the inbreeding coefficient *F*_IS_, which measures the reduction in heterozygosity due to inbreeding [37,38]. To assess the genetic relatedness of the populations, we calculated the average *F*_ST_ for pairwise comparisons of all sampled populations in the present study. We then used these average pairwise *F*_ST_ values to cluster populations by a neighbor-joining method implemented in the ***Mega*6.0** program [39].

To analyze the organization of the populations using multilocus genotypic information, the ***populations*** program in ***Stacks*** was used to output SNP data across all RAD sites into *Structure*-format files [40–42]to analyze the genetic structure at the population level and into *Genepop*-format files to estimate the gene flow among populations using the ***Genepop4*.*0*** software (http://genepop.curtin.edu.au/). During this data outputting process, only the first SNP per locus was written in both the *Genepop*-format and *Structure*-format outputs to avoid tight linkage SNPs [35]with the output parameters r = 1 and p = 6.

The distribution of genetic variation was analyzed by AMOVA analysis using the ***Arlequin3*.*5*** software [43] after converting the *Genepop*-format files into an *Arlequin*-compatible format.

Sample assignment analysis was performed using the software ***Structure2*.*3*** [40] on the complete data produced by the ***populations*** program. For this analysis, 10000 burn-in steps and 100000 iterations were used, with 10 replicates for each value of K, where K is the number of genotypic groups, which ranged from 1 to 12. Output data were processed in ***Structure Harvester v0*.*693*** [44] (http://taylor0.biology.ucla.edu/structureHarvester/). The optimal K for each analysis was chosen using the delta K method of Evanno et al. [45], as implemented in ***Structure Harvester***. Genetic relationships among the studied individuals were also assessed by principal coordinates analysis (PCoA) in R software package ***adegenet*** (http://adegenet.r-forge.r-project.org/files/montpellier/practical-MVAintro.1.0.pdf). based on the Euclidian distances between individual genotypes.

Gene flow (*Nm*) at the species level was estimated using the software ***Genepop4*.*0*** (http://genepop.curtin.edu.au/), and the pairwise *Nm* values at the population level were measured using the formula *Nm* = (1—*F*_ST_) / 4 *F*_ST_[46,47] and based on the *F*_ST_ values derived from the ***populations*** program.

To estimate the migration rates and effective population size for Guangxi and Yunnan groups as well as ancestral group, IM model was performed using ***IMa*** [48]. The *Phylip*–format SNP data was output by ***populations*** with the parameters p = 1 and r = 24, in which one locus was present in at least 24 individuals was used as the input file after manually editing. Demographic parameters including effective population sizes (θ_1_, θ_2,_ and θ_A_) and migration rates (m_1_ and m_2_) were estimated by 20000000 steps following 200000 burn-in periods. To verify convergence upon the same values, the analysis was repeated three times using the same priors but different seeds in each one of the runs.

## Results

### Sequence data quality and processing

The raw sequence data of most samples were around or greater than 1000Mb, whereas three samples had less than 700 Mb of raw data. After filtering by ***process_radtags***, the clean data derived from each sample ranged from 595Mb to 1880Mb, and most of them were approximately 1000Mb (S1 Table). All of the clean data were of high quality, as assessed by ***FastQC*** (http://www.bioinformatics.babraham.ac.uk/projects/fastqc/). The loci of each sample were produced after clean data processing analysis done using the ***ustacks***-***cstacks***-***sstacks*** program (S1 Table).

To evaluate the genetic diversity of *P*. *notoginseng* at the species level using ***populations***, all 36 samples were treated as a whole population. After requiring loci to be present in at least 67% of samples, 25543 RAD loci were retained. Each plantation was treated as a population when evaluating genetic diversity at the population level. After requiring loci to be present in all individuals of at least six populations, 13216 loci were retained.

### Genetic diversity at the species and population (plantation) levels

For all loci that were polymorphic in the entire data set at the species level, the observed heterozygosity (*H*_o_) was 0.1523, the expected heterozygosity (*H*_e_) was 0.1554, the nucleotide diversity (*π*) was 0.159, and the inbreeding coefficient (*F*_IS_) was 0.0591. When considering all nucleotide positions, including the non-polymorphic ones, the observed heterozygosity decreased to 0.0016, the expected heterozygosity to 0.0016, the nucleotide diversity to 0.0017, and the inbreeding coefficient to 0.0006.

**Fig 2:**
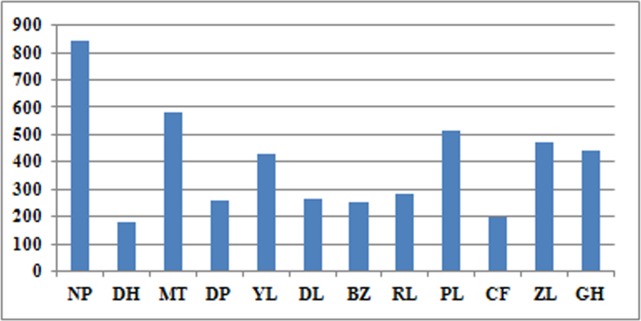
The distribution of private allele numbers among populations with p = 6/r = 1.

**Fig 3:**
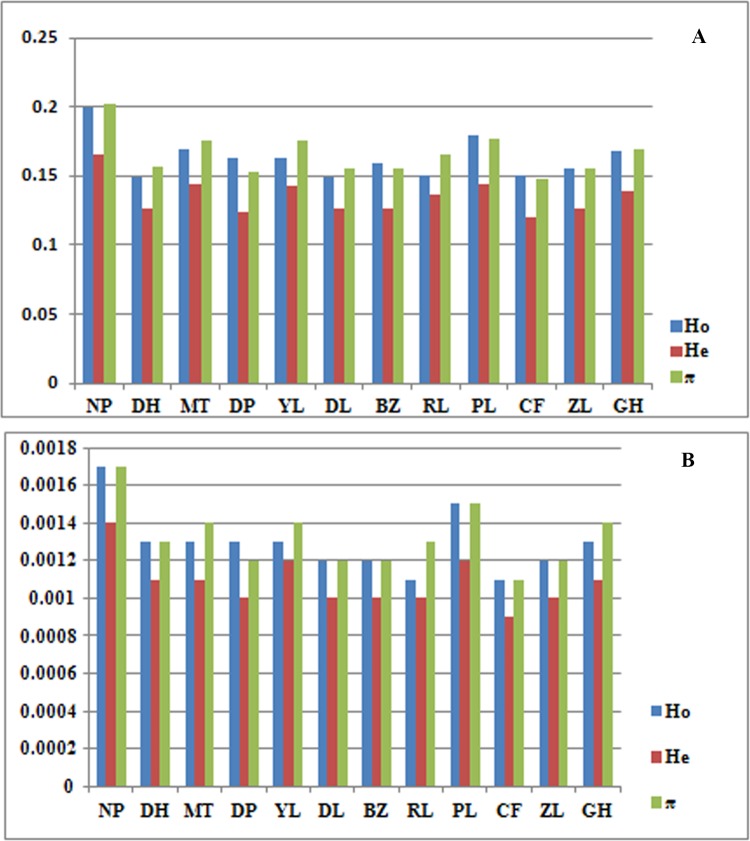
Distribution of genetic diversity indices, including observed heterozygosity (*H*_o_), expected heterozygosity (*H*_e_) and nucleotide diversity (*π*) with p = 6/r = 1. (**A**)Genetic diversity indices were based on variant position data, and on (**B**) all position data.

The statistics for each population were shown in Table 1 and Figs 2 and 3. For all loci that were polymorphic in at least one population in the entire data set, the average observed heterozygosity ranged from 0.1489 to 0.1997, the expected heterozygosity ranged from 0.1197 to 0.1650, the nucleotide diversity ranged from 0.1473 to 0.2020, and the inbreeding coefficient ranged from -0.0114 to 0.0282. When considering all nucleotide positions, including the non-polymorphic ones anywhere in the dataset, the observed heterozygosity decreased to 0.0011 to 0.0017, the expected heterozygosity decreased to 0.0009 to 0.0014, the nucleotide diversity ranged from 0.0011 to 0.0017, and the inbreeding coefficient ranged from -0.0001 to 0.0002. The private allele number of each population ranged from 182 to 824. As indicated in Table 1and Figs 2 and 3, the NP population showed the highest genetic diversity, as revealed by the observed heterozygosity (*H*_o_), the expected heterozygosity (*H*_e_), the nucleotide diversity (*π*) and the private allele numbers. The MT and PL populations had relatively higher genetic diversity than did the other populations. In contrast, the CF population showed the lowest heterozygosity and nucleotide diversity values, and the DH population had the fewest private alleles.

**Table 1:** The statistical values of genetic diversity within populations from variant and all positions data with p = 6/r = 1.

Pop	Private		*H*_o_		*H*_e_		*π*		*F*_IS_
code		variant positions	all positions	variant positions	all positions	variant positions	all positions	variant positions	all positions
NP	824	0.1997	0.0017	0.1650	0.0014	0.2020	0.0017	0.0045	0.0000
DH	182	0.1489	0.0013	0.1267	0.0011	0.1571	0.0013	0.0156	0.0001
MT	581	0.1691	0.0013	0.1436	0.0011	0.1750	0.0014	0.0116	0.0001
DP	259	0.1625	0.0013	0.1244	0.0010	0.1531	0.0012	-0.0114	-0.0001
YL	432	0.1628	0.0013	0.1434	0.0012	0.1755	0.0014	0.0256	0.0002
DL	268	0.1492	0.0012	0.1261	0.0010	0.1557	0.0012	0.0140	0.0001
BZ	252	0.1586	0.0012	0.1266	0.0010	0.1560	0.0012	-0.0039	0.0000
RL	286	0.1504	0.0011	0.1361	0.0010	0.1660	0.0013	0.0282	0.0002
PL	517	0.1796	0.0015	0.1444	0.0012	0.1767	0.0015	-0.0038	-0.0000
CF	199	0.1508	0.0011	0.1197	0.0009	0.1473	0.0011	-0.0050	-0.0000
ZL	472	0.1560	0.0012	0.1267	0.0010	0.1550	0.0012	-0.0007	-0.0000
GH	444	0.1681	0.0013	0.1393	0.0011	0.1693	0.0014	0.0045	0.0000

Note: private, private allele number; *H*_o_, observed heterozygosity; *H*_e_, expected heterozygosity; *π*, nucleotide diversity; *F*_IS_, inbreeding coefficient of an individual relative to the subpopulation.

### Genetic distances within and among populations

When considering all polymorphic loci, the average *F*_IS_ values were very close to zero (ranging from -0.0114 to 0.0282 as shown in Table 1), thus indicating a lack of genetic structure or assortative mating within populations [30].

**Fig 4:**
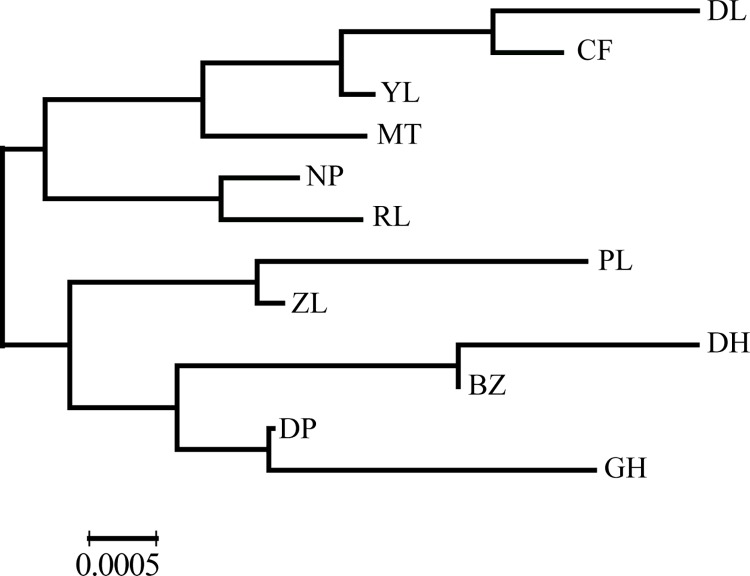
A neighbor-joining (NJ) tree created using pairwise *F*_ST_ values as distance metrics with p = 6/r = 1.

The *F*_ST_ values shown in Table 2 were very close to zero and therefore may not be biologically significant, indicating that there was nearly no differentiation among populations of *P*. *notoginseng*. The neighbor-joining tree based on the *F*_ST_ values revealed that three populations (NP, RL and CF) from the Guangxi province grouped into a branch (Fig 4).

**Table 2:** Pairwise comparison of genetic distances (*F*_ST_ values) among *P*. *notoginseng* populations with p = 6/r = 1.

Pop code	DH	MT	DP	YL	DL	BZ	RL	PL	CF	ZL	GH
NP	0.0016	0.0043	0.0080	0.0056	0.0041	0.0057	0.0023	0.0080	0.0020	0.0050	0.0128
DH		0.0063	0.0039	0.0074	0.0026	0.0058	0.0016	0.0063	0.0024	0.0053	0.0121
MT			0.0077	0.0115	0.0080	0.0059	0.0080	0.0051	0.0040	0.0016	0.0129
DP				0.0036	0.0056	0.0056	0.0025	0.0072	0.0077	0.0065	0.0175
YL					0.0060	0.0046	0.0022	0.0020	0.0032	0.0053	0.0114
DL						0.0059	0.0030	0.0033	0.0017	0.0034	0.0193
BZ							0.0047	0.0027	0.0023	0.0021	0.0047
RL								0.0072	0.0067	0.0025	0.0099
PL									0.0024	0.0048	0.0063
CF										0.0056	0.0075
ZL											0.0087

### Population structure and gene flow

We used an AMOVA approach to more precisely partition the genetic variation across populations with 6418 SNPs that were produced by ***Stacks*** (see description in methods). Most (~96.5%) of the genetic variation occurred within populations, whereas only approximately 3.5% of the variation occurred among populations(Table 3). These results confirmed the conclusions deduced from the *F*_ST_ analysis.

**Table 3:** The results of the AMOVA analysis.

Source of variation	Sum of squares	Variance components	Percentage variation (%)
Among populations	1.597	0.00429	3.46939
Within populations	7.167	0.11944	96.53061
Total	8.764	0.12374	

**Fig 5:**
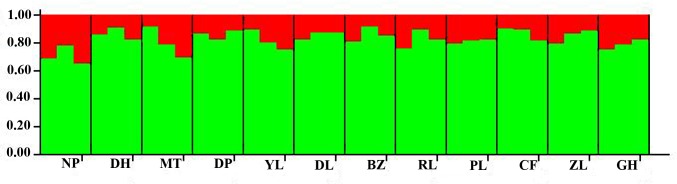
Bayesian inference of the number of clusters (K) of *Panax notoginseng* based on *Structure* analysis.

**Fig 6:**
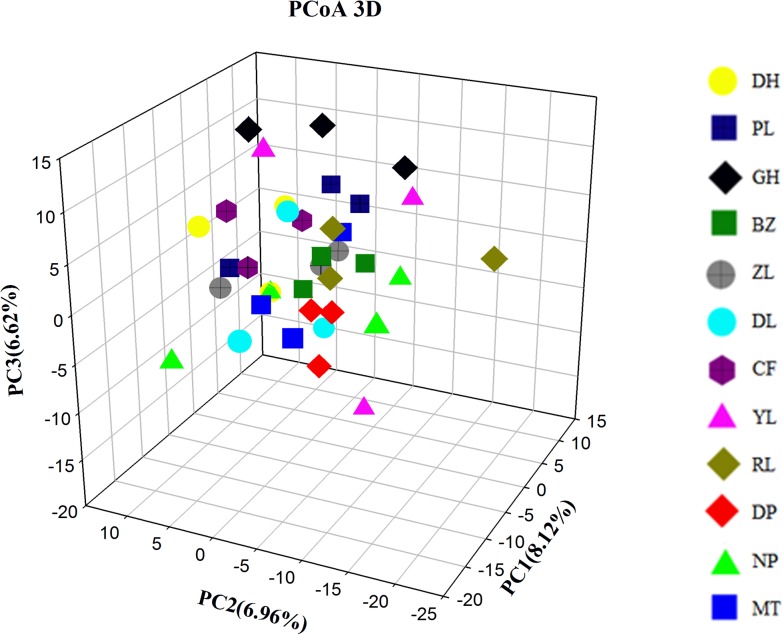
Principal coordinates analysis (PCoA) plot generated by *adegenet*.

As a further test of potential population structure, we analyzed these 6418 SNPs using the software ***Structure2*.*3*** [40] with an “admixture model” and a “correlated alleles frequencies model”. Because loci in tight linkage should be avoided in ***Structure*** analyses, only one SNP was chosen from each RAD site, which means that these 6418 SNPs came from 6418 RAD sites. By examining the change in LnP(D), and using the deltaK approach of Evanno [45], we found that a model with K = 2 best fits the data (S1 and S2 Figs). However, an examination of the posterior probabilities plot (Fig 5) did not show two distinct clusters; all samples were genetically intermingled and had admixed ancestry. Principal coordinates analysis did not produce any distinct groupings either (Fig 6), which was consistent with the results of ***Structure*** analysis. These results further supported the hypothesis that no genetic differentiation occurred among Sanqi populations.

***Genepop*** analyses revealed that the overall number of migrants (*Nm*) per generation was 1.4. The pairwise population *Nm* values calculated from Wright’s analysis indicated that the level of gene flow between populations was substantially high, with the largest being 156.0 between the NP and DH populations and the smallest being 12.7 between the GH and DL populations.

### Demographic parameters estimated using the IM model

The demographic parameters of IMa analysis are shown in Table 4, and the marginal distributions of the probabilities of the parameters are shown in supporting file S3. The effective population size of Guangxi group (θ_1_) was smaller than that of Yunnan group (θ_2_), and both were much smaller than that of the ancestral population (θ_A_). The migration rates between the Guangxi and Yunnan groups (m1 and m2) were not significant.

**Table 4:** The demographic parameters estimated using IM model.

Value	θ_1_	θ_2_	θ_A_	m1	m2
HiPt	0.6683	2.1004	1770.7055	0.0010	0.0015
HiSmith	0.7320	2.1004	1770.7055	0.0010	0.0015
HPD90Lo	0.0955	0.4455	1376.0838	0.0010	0.0015
HPD90Hi	3.1506	7.8288	2330.8136	1.7050	2.0265

## Discussion

### Low genetic diversity of *P*. *notoginseng*

Nucleotide diversity (*π*)is known as the average pairwise difference between two DNA sequences, and it is a measure of expected heterozygosity for bi-allelic SNP markers [30,49]. Different DNA fragments in one species may not have the same π values due to variation in evolutionary rates [50]. Shi et al.[18] selected 36 single-copy nuclear genes to infer the phylogenetic relationships of the *Panax* species and evaluate whether the same ortholog exhibits heterogeneous evolutionary rates in diploid and tetraploid species. The π values of *P*. *notoginseng* ranged from zero to 0.0139, with anaverage value of 0.0045,whereas the tetraploid *P*. *ginseng* and *P*. *quinquefolius* had an average π value of 0.0097 and 0.0104, respectively. The π values of these selected genes are not more representative than those from RAD tags when used to describe the genetic diversity of a species. Our study revealed that the π values *P*. *notoginseng* estimated using RAD sequencing data were 0.0017 at a species-wide level and 0.0011 ~ 0.0017 at a population level. These data indicated that the nucleotide diversity level of Sanqi was low. Using RAD tags, Xiao [51] reported the genetic diversity of *Phoebe zhennan*, an endemic and endangered species in China. The π values of this tree species ranged from 0.0010 to 0.0016 among different populations[51], which is very similar to the estimated Sanqi range. The genomic nucleotide diversity of cultivated soybean (*Glycine max*) was 0.0005 ~ 0.0010[52], as estimated by SLAF-seq, which is another reduced-representation sequencing technology similar to RAD sequencing [53]; the estimated values were even lower than those of *P*. *notoginseng* and *P*. *zhennan*.

Heterozygosity(*H*), including observed heterozygosity(*H*_o_) and expected heterozygosity(*H*_e_), is another important statistic in population genetics. Although the heterozygosity values estimated using different DNA markers will vary in plants [54], we made a comparison of the heterozygosity values estimated with different markers in *P*. *notoginseng* and two relatives, *P*. *ginseng* and *P*. *quinquefolius*. The *H*_e_ value of *P*. *notoginseng* estimated from polymorphic sites using RAD sequencing was 0.1554. The total genetic diversity of wild *P*. *ginseng* estimated from allozyme data was low at the species level (*H*_e_ = 0.0230) [55], but its average expected heterozygosity estimated using the RAPD method was 0.1348 [56]. For cultivated populations of *P*. *quinquefolius*, the *H*_e_ values were 0.1637 based on RAPD data [56] and 0.3100 based on allozyme data [57].

The effective size (*N*_e_)of a population, reflecting the rate at which genetic diversity will be lost, will be reduced by a population bottleneck [46]. The IM analysis revealed that the effective population sizes of Guangxi group (θ_1_) and Yunnan group (θ_2_) were both much smaller than that of the ancestral population (θ_A_), which meaning that serious population contraction has occurred in the two distribution areas of Sanqi.

All the findings mentioned above indicated that *P*. *notoginseng* probably experienced domestication bottlenecks [58,59] and thus lost a certain amount of genetic diversity. This bottleneck probably occurred in the early domestication process when only a limited number of individuals of the progenitor species were used by the early farmers, which left most of the genetic diversity in the progenitor behind. During the subsequent cultivation process, weak artificial selection for special agronomic traits has been carried out in Sanqi, but only seeds from the strongest plants in each generation were chosen to give rise to the next generation. This winnowing can also cause a severe loss of genetic diversity [3,58]. *Scrophularia singpoensis* is a famous medicinal plant in China with a domestication history about 1000 years. The cpDNA nucleotide diversity is 0.00076 and 0.00301 of cultivated and wild populations, respectively [60]. Genetic diversity of the cultivated species is usually low whether it has been domesticated for a long time or not [60–63].

### Almost no genetic differentiation among populations

Genetic differentiation usually results from a long evolutionary period and is affected by biological features, such as mating systems, life history traits and gene flow. The differentiation index (*F*_ST_) among Sanqi populations ranged from 0.0016 to 0.0193, which is significantly lower than the average *F*_ST_ values of species with mixed mating systems or with short-lived perennial history [54], suggesting that all populations of Sanqi were genetically similar. Wright [64] explained that if *F*_ST <_ 0.0500, there is almost no differentiation between populations. Furthermore, AMOVA analysis revealed that only 3.47% of the genetic variation occurred between populations and that approximately 96.5% of the genetic variation occurred within populations. The results of the ***Structure*** analysis and Principal Coordinates Analysis also supported this conclusion. Wang et al. [65] analyzed the chemical variation of *P*. *notoginseng* and found no significant differences in saponin concentration among different groups; however, the saponin concentration exhibited great variation among individual samples. This distribution pattern of chemical variation coincided with the pattern of genetic variation revealed in the present study. A mixed NJ tree estimated from 11713 bp further supported this conclusion (S4 Fig), too. Population structure is strongly influenced by genetic exchange patterns (gene flow) within and between populations [66], and only when the lack of gene flow occurs, will the mutation and genetic drift cause populations to genetically diverge from one another [46]. The overall gene flow of *P*. *notoginseng*, *Nm*, is> 1, suggesting that frequent genetic exchange among populations could hold back the genetic differentiation from occurring [67]. Similar to most medicinal plants, the strong gene flow in *P*. *notoginseng* comes from the frequent seed exchanges among different farms. In addition, there was a lack of breeding selection during the domestication process such that no cultivars or landraces have been created, and *P*. *notoginseng* is still a mixed population of individuals with heterogeneous phenotypic features such as red or yellow seeds and green or dark red stems [68,69]. This kind of lack of strong artificial selection on special agronomic traits is also the cause of the absence of genetic differentiation in Sanqi. In addition, Sanqi has relatively short cultivation history, and insufficient splitting time between populations should be a cause of the lack of differentiation, too [60]. Some cultivated medicinal plants usually have no genetic differentiation with most genetic variation occurring within populations even though they have longer cultivation history than Sanqi or have phenotypic or agro-ecological groups [70–72]. And the insufficient splitting time also resulted in the lack of the differentiation between the Guangxi group and Yunnan group although the migration rates (demographic parameters, m1 and m2) between them were not significant.

### Implications for the Conservation of Genetic Resources

Reduced genetic variation might restrict the fitness of domesticated individuals. *P*.*notoginseng* faces serious recurrent cropping obstacles. After a three-year cropping period, the farmers must wait for 7~10 years until the soil can be used to plant Sanqi again. In addition, as a medicinal plant, the planting scale of *P*. *notoginseng* is seriously influenced by the market demand. When the price is low, the number and size of plantations decrease quickly. For example, the Guangxi Zhuang Autonomous Region of China used to be the main *P*. *notoginseng* production district, and there were many plantations in the Jingxi, Napo and Debo counties of Guangxi before 1988 [73]. However, with the price of Sanqi falling sharply on a large scale in the late 1980s, most of the plantations have disappeared and only three populations (NP, RL and CF) were found in Jingxi county when we collected the materials used in the present study in 2011. Some genetic resources have likely been lost with the disappearance of most plantations. Therefore, germplasm nurseries and banks should be built as soon as possible to maintain and protect the existing genetic resources of *P*. *notoginseng*.

Although the genetic diversity of *P*. *notoginseng* was low at both the species and population levels, several traditional plantations such as NP and PL had a relatively higher genetic diversity level than did others based on *H*_e_ and *π* values as well as on the private allele numbers (shown in Table 1).The NP plantation is located in the Jingxi county of Guangxi, and the owners of this plantation have been cultivating Sanqi for approximately 40 years. It is likely that continuous cultivation has allowed large amounts of genetic variation to still be available today, though most such plantations disappeared in the 1980s. Furthermore, all of the seeds used to propagate the crops were collected from their own plantation during cultivation, which suggests that there was almost no seed flow between NP and other plantations. This cultivation model ensured that the NP population had the most private alleles. Thus, the NP plantation should be the first choice for us to collect the genetic resources for breeding or conservation.

In addition to the NP population, the PL plantation, which has a traditional Sanqi cultivation history, had high genetic diversity. This plantation, located in the Wenshan county of the Yunnan province, is a large planting base for a Sanqi production company. Many individuals of *P*. *notoginseng* with various excellent agricultural traits (e.g., purple roots) [68] have been collected from other plantations and planted in this one. In other words, the samples from this plantation not only harbored higher genetic diversity but also had the most variable agricultural traits. These valuable resources should be protected as soon as possible so they can be used in future breeding projects.

### The possible geographical origins of Sanqi domestication

The first historical record of Sanqi can be found in the “Compendium of Materia Medica”, a book on Chinese herbal medicine published in 1596. In this book, Sanqi is described to have been discovered in the mountains of west Guangxi. Approximately 150 years later, cultivated Sanqi has been shown to be sold in Wenshan, Yunnan, based on observations recorded in the “Annals of Kaihua Prefecture”, published in 1757. The possible geographical origin of Sanqi domestication is therefore still controversial [73]. Private alleles may provide evidence on the center of origin of this crop [62]. The results of the present study revealed that the NP population located in Guangxi had the highest private allele number, which suggests that the primitive domestication of Sanqi probably occurred first in Guangxi and then dispersed to Yunnan. Although Sanqi has been cultivated for just approximately 400 years, no wild resources can be found today. The wild individuals were probably driven to extinction by over harvesting in the past years. On the other hand, the cultivated Sanqi probably originated from the hybrid events of wild relative species, which could be deduced by its owning of admixed ancestry as revealed by structure analysis [74–79]. Fortunately, wild populations of several generic species such as *P*. *stipuleanatus*, *P*. *zingiberensis*, *P*. *japonicus* and *P*. *vietnamensis* still exist in Yunnan, which could potentially provide genetic resources for the improvement of cultivated Sanqi and the further research of Sanqi origin.

## Supporting Information

S1 Fig The mean posterior probability of each given K (10replicates)estimated from Bayesian analysis as implemented by *Structure*. (EPS)

S2 Fig The distribution of ΔK estimated from Bayesian analysis as implemented by *Structure*. (EPS)

S3 Fig Marginal distribution of the posterior probability of five demographic parameters estimated by the IM model. (EPS)

S4 Fig The NJ tree of *P*. *notoginseng*. (EPS)

S1 Table Summary statistics for each RAD-sequencing data sample, including reads length, clean read number, base count and the loci number matched to the catalog produced by *Stacks*. (DOCX)
