# Supplementary material for: Application of RAD Sequencing for Evaluating the Genetic Diversity of Domesticated Panax notoginseng (Araliaceae)
Source: PLoS One. 2016 Nov 15;11(11):e0166419. doi: 10.1371/journal.pone.0166419 (PMC5112861; doi:10.1371/journal.pone.0166419)
Supplement: S1 Table — (DOCX) [file pone.0166419.s005.docx]

S1 Table. Summary statistics for each sample of RAD-sequencing data processing, including the reads length, clean read number, base count and the loci number matched to the catalog produced by ***Stacks***

| Sample code | Read length | Clean read count | Clean data (base count) | Loci count matched |
| --- | --- | --- | --- | --- |
| NP-1 | 93-95 | 11358924 | 1067738856 | 201115 |
| NP-2 | 95 | 8653201 | 822054095 | 181704 |
| NP-3 | 93-95 | 11602955 | 1090677770 | 172943 |
| DH-1 | 94 | 13960893 | 1312323942 | 180946 |
| DH-2 | 94 | 9830321 | 924050174 | 306181 |
| DH-3 | 94 | 11557513 | 1086406222 | 245157 |
| MT-1 | 93 | 12176957 | 1132457001 | 143243 |
| MT-2 | 93 | 13227885 | 1230193305 | 161605 |
| MT-3 | 93 | 12542296 | 1166433528 | 193620 |
| DP-1 | 93 | 12285476 | 1142549268 | 166372 |
| DP-2 | 93 | 12106359 | 1125891387 | 154573 |
| DP-3 | 95 | 10540698 | 1001366310 | 164179 |
| YL-1 | 96 | 9635219 | 924981024 | 302502 |
| YL-2 | 95 | 9740548 | 925352060 | 272820 |
| YL-3 | 95 | 15784439 | 1499521705 | 415878 |
| DL-1 | 96 | 6196675 | 594880800 | 243990 |
| DL-2 | 96 | 7157204 | 687091584 | 102882 |
| DL-3 | 97 | 10443875 | 1013055875 | 110982 |
| BZ-1 | 95 | 12438217 | 1181630615 | 183321 |
| BZ-2 | 96 | 10734931 | 1030553376 | 251505 |
| BZ-3 | 96 | 8087648 | 776414208 | 256593 |
| RL-1 | 95 | 10822619 | 1028148805 | 218080 |
| RL-2 | 95 | 14880731 | 1413669445 | 167008 |
| RL-3 | 96 | 11911631 | 1143516576 | 265366 |
| PL-1 | 94 | 9440785 | 887433790 | 151015 |
| PL-2 | 94 | 12470435 | 1172220890 | 210609 |
| PL-3 | 94 | 14801418 | 1391333292 | 409174 |
| CF-1 | 96 | 8906575 | 855031200 | 97816 |
| CF-2 | 96 | 11462609 | 1100410464 | 141999 |
| CF-3 | 96 | 14205730 | 1363750080 | 272549 |
| ZL-1 | 95 | 7010102 | 665959690 | 225131 |
| ZL-2 | 96 | 9901678 | 950561088 | 263064 |
| ZL-3 | 95 | 12164272 | 1155605840 | 119366 |
| GH-1 | 94 | 14625562 | 1374802828 | 213833 |
| GH-2 | 94 | 12079724 | 1135494056 | 203674 |
| GH-3 | 94 | 19986531 | 1878733914 | 370491 |
